# Haematological Tracking of Thyroid Status During Methimazole Therapy and After Treatment Interruption in Hyperthyroid Cats: A Prospective Longitudinal Cohort Study

**DOI:** 10.3390/vetsci13070706

**Published:** 2026-07-18

**Authors:** Andrei Răzvan Codea, Alexandra Biriș, Sidonia Gog-Bogdan, Alina Diana Haşaş, Daniela Neagu, Cristian Popovici, Mircea Mircean

**Affiliations:** 1 Department of Internal Medicine, Faculty of Veterinary Medicine, University of Agricultural Sciences and Veterinary Medicine Cluj-Napoca, Calea Mănăştur 3–5, 400372 Cluj-Napoca, Romania; alexandra.biris@usamvcluj.ro (A.B.); alina.hasas@usamvcluj.ro (A.D.H.); daniela.neagu@usamvcluj.ro (D.N.); cristian.popovici@usamvcluj.ro (C.P.); mircea.mircean@usamvcluj.ro (M.M.); 2 Department of Surgery, Faculty of Veterinary Medicine, University of Agricultural Sciences and Veterinary Medicine Cluj-Napoca, Calea Mănăştur 3–5, 400372 Cluj-Napoca, Romania

**Keywords:** feline hyperthyroidism, methimazole, haematocrit, mean corpuscular volume, complete blood count, total thyroxine, therapeutic monitoring, longitudinal study, ROC analysis, erythropoiesis

## Abstract

**Background:** Feline hyperthyroidism is the most prevalent endocrine disorder of older cats; TT4 monitoring is costly and often inaccessible. **Methods:** Forty-three cats with primary hyperthyroidism were followed prospectively at baseline (T0) and at 1, 3 and 6 months (T1, T2, T3). Hct, Hgb, MCV, ALT and TT4 were measured at each visit. Per-timepoint correlations, repeated-measures correlation (rmcorr), linear mixed-effects models, and Friedman and ROC analyses were performed. **Results:** TT4 correlated with Hct (r = 0.875 at T1 to 0.943 at T3), MCV (0.907 to 0.964), Hgb (0.836 to 0.946) and ALT (0.919 to 0.981), intensifying progressively. Within individual cats, Hct (rmcorr r = 0.873) and MCV (rmcorr r = 0.881) tracked TT4, and for Hct, the within-cat coefficient exceeded the between-cat coefficient. All primary parameters and ALT changed significantly from T0 to T1 (all *p* < 0.001). ROC analysis identified Hct ≥ 35.2% at T2 as the optimal cut-off (AUC = 0.972, 95% CI 0.914–1.000; sensitivity 96.7%, specificity 92.3%). **Conclusions:** Hct and MCV tracked TT4 within individual cats during methimazole therapy and returned toward pre-treatment values after discontinuation. A rising Hct or MCV in a treated cat may signal loss of therapeutic control and prompt TT4 re-evaluation, without replacing hormonal monitoring.

## Simple Summary

Hyperthyroidism is the most common hormonal disease in older cats and requires lifelong treatment with methimazole, a drug that suppresses thyroid hormone production. Monitoring treatment effectiveness currently relies on repeated blood tests measuring thyroid hormone levels (T4), which can be expensive and difficult to access in many veterinary practices. This study investigated whether widely available blood count parameters, particularly the proportion of red blood cells (haematocrit) and their average size (mean corpuscular volume), change in parallel with thyroid hormone levels during treatment and after treatment is interrupted. In 43 hyperthyroid cats followed over six months, both parameters dropped significantly as T4 normalised and rose again when treatment was interrupted, closely mirroring thyroid hormone levels. A haematocrit at or above 35.2% at the three-month check-up correctly identified cats with inadequately controlled hyperthyroidism in 96.7% of cases in this referral cohort; performance at lower disease prevalence will require prospective validation. These findings suggest that veterinarians could use a routine blood count result as a low-cost early-warning signal between scheduled thyroid hormone tests, prompting a re-evaluation when the haematocrit or mean corpuscular volume rises toward or above pre-treatment levels in a cat receiving methimazole.

## 1. Introduction

Feline hyperthyroidism is the most prevalent endocrine disorder of domestic cats, first described by Holzworth et al. in 1980 [1] and subsequently characterised by Peterson et al. in 131 cases [2]. Prevalence has increased markedly over the past four decades, attributable to improved diagnostic access, increased longevity and possibly environmental and dietary factors [3,4,5,6]. The condition results from autonomous secretion of T4 and T3 by adenomatous thyroid tissue, producing a multisystemic syndrome of weight loss, polyphagia, tachycardia and hypertension, with associated structural cardiac changes [7,8,9,10,11,12]. Diagnosis is confirmed by elevated TT4, with free T4 and thyroid-stimulating hormone assays used as adjuncts in equivocal cases [9].

Methimazole, a thioamide that inhibits thyroid peroxidase, is the most widely used medical treatment for feline hyperthyroidism [13,14]. Lifelong administration requires regular monitoring to maintain euthyroidism, prevent iatrogenic hypothyroidism and detect treatment failure [15,16]. Current guidelines typically recommend TT4 measurement at 2–3 weeks, 4–6 weeks, 3 months and every 6 months thereafter [9,13,17]. In practices with limited endocrinological laboratory access, particularly in Central and Eastern Europe, this schedule is not consistently observed, leading to suboptimal therapeutic management. A survey of UK and international practitioners confirmed that monitoring schedules are inconsistently applied in clinical practice, with significant variation in target TT4 levels and recheck intervals [18]. Owner compliance with monitoring schedules has also been identified as a significant barrier to optimal therapeutic management in referral populations [19]. The haematological and clinical presentation of feline hyperthyroidism, including stress leukograms and erythrocytosis, has been well characterised in published case series [2,20].

Thyroid hormones are key regulators of erythropoiesis, stimulating renal erythropoietin secretion, amplifying erythroid progenitor sensitivity and accelerating marrow maturation, as demonstrated in experimental in vitro models [21,22,23]. In human medicine, hyperthyroidism is consistently associated with erythrocytosis and macrocytosis, whereas hypothyroidism produces normocytic or microcytic anaemia [24,25]. In cats, the haematocrit and MCV may be in the upper reference range or mildly elevated at diagnosis in some patients; however, the haematological presentation is heterogeneous, with erythrocytosis documented in approximately 13% of hyperthyroid cats and microcytosis in up to 30% of those referred for radioiodine treatment [26,27]. Hepcidin, an iron-regulatory peptide that restricts iron availability for erythropoiesis, has been proposed as a mechanistic link between thyroid hormone excess and alterations in erythrocyte indices in hyperthyroid patients; hepcidin concentrations have been shown to decline in parallel with TT4 normalisation, with potential implications for erythrocyte index variability in patients with more severe or prolonged thyroid hormone excess [28,29]. The longitudinal kinetics of haematological parameters relative to TT4 during methimazole therapy, and their discriminatory utility as surrogate monitoring tools, have not been prospectively evaluated. We hypothesised that the haematocrit and MCV may track TT4 bidirectionally during methimazole therapy and following loss of therapeutic control, and that their dynamics are sufficiently consistent to discriminate controlled from uncontrolled hyperthyroidism at routine monitoring visits.

The primary objective of this study was to characterise the correlation between TT4 and haematological parameters (Hct, Hgb, MCV) and ALT at each monitoring timepoint over six months of methimazole therapy in a prospective cohort stratified by therapeutic response. Secondary objectives were to evaluate bidirectional haematological dynamics following loss of therapeutic control and to determine optimal haematological cut-offs for identifying uncontrolled hyperthyroidism by ROC analysis.

## 2. Materials and Methods

### 2.1. Study Design and Population

A prospective longitudinal observational study was conducted at the Internal Medicine Department, Faculty of Veterinary Medicine, USAMV Cluj-Napoca, Romania, between August 2024 and March 2026. Forty-three client-owned domestic cats with confirmed primary spontaneous hyperthyroidism were enrolled. The eligibility criteria, particularly the exclusion of cats with concurrent conditions known to independently affect haematological parameters, resulted in a screening-to-enrolment ratio of approximately 3:1, with the majority of screened cats excluded due to concurrent CKD IRIS ≥ 2, neoplasia or owner non-availability for follow-up. Ethical approval for this study was obtained from the Bioethics Committee of USAMV Cluj-Napoca after data collection had been completed (application no. 557 of 8 June 2026; Decision no. 570 of 12 June 2026). The authors acknowledge that approval was not sought before enrolment of the first patient. This study was initiated as a structured clinical follow-up of client-owned cats presenting for the routine diagnosis and management of hyperthyroidism, and all sampling, treatment and monitoring procedures corresponded to the standard of care recommended for this condition. No procedure was performed for research purposes alone, no additional blood volume was collected, and no cat was subjected to any intervention it would not otherwise have received. The Bioethics Committee reviewed the study as submitted and issued a favourable opinion, valid for the entire duration of the study. All procedures were performed exclusively within the routine diagnostic and therapeutic care of client-owned animals, without additional interventions beyond the standard of care. Written informed consent was obtained from all cat owners prior to enrolment.

### 2.2. Inclusion and Exclusion Criteria

Inclusion criteria were: (i) age ≥ 8 years; (ii) TT4 > 4.0 µg/dL by ELISA; (iii) compatible clinical signs; (iv) naïve to antithyroid treatment; (v) absence of conditions independently affecting haematological parameters (CKD IRIS ≥ 2, neoplasia, inflammatory disease, anaemia of other aetiology defined as anaemia attributable to causes independent of thyroid hormone excess). Inflammatory disease was defined at baseline as any clinically apparent inflammatory or infectious condition identified on history and physical examination (including pyrexia, chronic gingivostomatitis, upper respiratory tract disease, dermatitis, abscessation or clinically evident inflammatory bowel disease), or a baseline leucocyte count outside the reference interval. Cats meeting either criterion were not enrolled. Cats with owners unwilling to comply with the follow-up schedule or with a baseline body weight < 2.0 kg were excluded [9]. Cats with CKD IRIS stage ≥ 2 were specifically excluded because renal-associated anaemia may independently reduce the haematocrit, confounding the primary TT4-Hct relationship under investigation.

### 2.3. Diagnostic Protocol and Therapeutic Groups

Diagnosis was confirmed by physical examination, thyroid palpation, and systolic blood pressure measurement (Doppler). TT4 was measured by a competitive ELISA using a commercial Cat Thyroxine (T4) ELISA Kit (Catalog No. CK-bio-20679, Shanghai Coon Koon Biotech Co., Ltd., Shanghai, China) validated for use in feline serum according to manufacturer specifications, read on a microplate photometer (HiPo MPP-96, Biosan, Riga, Latvia) and a thermostatic shaker-incubator (PST-60HL-4, Biosan, Riga, Latvia). All cats received methimazole (Felimazole^®^, Dechra, Boston, MA, USA) at 2.5 mg/cat twice daily, titrated to maintain TT4 within the reference range (1.5–4.0 µg/dL) [9,13].

Based on the six-month therapeutic response, cats were retrospectively categorized into: Group A (*n* = 13, sustained euthyroidism at all post-treatment timepoints; 13/13 always controlled); Group B (*n* = 8, uncontrolled at T1 or T2 requiring dose titration; 7/8 never achieved sustained control, and one cat achieved transient euthyroidism at T3 only); Group C (*n* = 11, initial biochemical response at T1 followed by loss of control due to owner-initiated treatment discontinuation representing a behavioural rather than pharmacodynamic failure mode); Group D (*n* = 11, primary non-responders in whom TT4 did not decrease below 4.0 µg/dL despite dose titration; contributing factors could not be formally assessed in this study). In the event of ambiguous therapeutic trajectories, cats were assigned to the group best representing the predominant pattern over the six-month period. This stratification was used for descriptive subgroup analyses only.

### 2.4. Sampling and Laboratory Methods

Blood samples were collected at T0 (baseline), T1 (30 ± 5 days), T2 (90 ± 7 days) and T3 (180 ± 14 days). Venous blood (1 mL EDTA, 2 mL plain) was collected by cephalic or jugular venipuncture. Complete blood count was analysed by an automated veterinary haematology analyser (LaserCyte Dx, IDEXX Laboratories, Westbrook, ME, USA). Parameters recorded were: Hct (%); Hgb (g/dL); MCV (fL); MCHC (g/dL); WBC (×10^9^/L); neutrophils (×10^9^/L); and platelets (×10^9^/L). MCHC was recorded at T0 only and was not analysed longitudinally, as it lacks direct mechanistic relevance to thyroid hormone-driven erythropoiesis. The haematology analyser generated a complete blood count at every visit. However, only the parameters pre-specified for this study (Hct, Hgb, MCV and reticulocytes) were transcribed into the study database at T1, T2 and T3. Leucocyte, neutrophil and platelet counts were transcribed at T0 only, since longitudinal leucocyte monitoring was not among the pre-specified objectives; the baseline leucocyte count was retained to characterise the stress leukogram and its correlation with TT4 at presentation, which is reported descriptively in the Results. The original analyser reports could not be retrieved retrospectively, and longitudinal leucocyte, neutrophil and platelet data are therefore not available for analysis. This limitation is addressed in the Limitations Section. Reticulocyte count (×10^9^/L) was additionally recorded at all four timepoints using the automated reticulocyte channel of the haematology analyser and analysed as a pre-specified secondary objective. Symmetric dimethylarginine (SDMA; µg/dL) was measured at all four timepoints using a point-of-care biochemical analyser (Catalyst One, IDEXX Laboratories, Westbrook, ME, USA) as a surrogate marker of the glomerular filtration rate to prospectively monitor renal function throughout the study period. TT4 was measured by ELISA and ALT by automated spectrophotometry (Catalyst One, IDEXX Laboratories, Westbrook, ME, USA). Haematological and TT4 measurements were performed simultaneously or within 48 h at each timepoint.

### 2.5. Statistical Analysis

Statistical analyses were performed using Python (version 3.12; Python Software Foundation, Wilmington, DE, USA) with the scipy (v1.11), scikit-learn (v1.3), statsmodels (v0.14) and pingouin (v0.6) libraries. Normality was assessed by Shapiro–Wilk test. Friedman’s test was used for repeated-measures comparisons, followed by Wilcoxon signed-rank post hoc tests with Bonferroni correction (α = 0.0083 for 6 pairwise comparisons). Pearson correlation coefficients (r) were calculated separately at each timepoint (*n* = 43 per timepoint) to avoid pseudo-replication inherent in pooled repeated-measures data; this approach preserves the independence assumption of Pearson’s test. Pooled correlations are reported for reference only and should be interpreted with caution given the non-independence of observations within repeated-measures data. To account for the non-independence of repeated measurements in longitudinal comparisons, Friedman’s test and Wilcoxon signed-rank post hoc tests were used exclusively for within-subject evolution; pooled Pearson correlations are presented as a descriptive complement to the per-timepoint analyses. The primary hypothesis concerned pre-specified correlations (TT4 vs. Hct and MCV); Bonferroni correction was applied to all post hoc pairwise comparisons. Although Shapiro–Wilk testing identified non-normality for some parameters at individual timepoints, Pearson correlations were retained as the primary correlation measure given the sample size (*n* = 43 per timepoint), which confers sufficient robustness under the central limit theorem; Spearman rank correlation coefficients were additionally computed at every time point and are reported in full alongside the per-timepoint Pearson coefficients. For the primary parameters (Hct, Hgb, MCV and ALT), they confirmed the direction and the statistical significance observed with Pearson’s r, with absolute differences between the two coefficients not exceeding 0.15. The only discordance concerned reticulocytes at T0, where the Pearson coefficient was nominally significant (r = 0.369, *p* = 0.015), whereas the Spearman coefficient was not (ρ = 0.268, *p* = 0.082). Results for secondary parameters with *p*-values between 0.0083 and 0.05 are reported descriptively and should be interpreted as hypothesis-generating rather than confirmatory. ROC analysis identified optimal cut-offs by the Youden index (J = sensitivity + specificity − 1) [30]. Bootstrap 95% confidence intervals for the AUC were computed using 2000 iterations (bias-corrected percentile method [31]). The binary outcome for the ROC was defined as TT4 > 4.0 µg/dL (uncontrolled hyperthyroidism); at T1, 19/43 cats (44.2%) were uncontrolled, at T2, 30/43 (69.8%) cats were uncontrolled, and at T3, 29/43 (67.4%) cats were uncontrolled. No cat developed iatrogenic hypothyroidism (TT4 < 1.5 µg/dL). Statistical significance was set at *p* < 0.05.

To address the repeated-measures structure of the data directly, two within-subject analyses were performed. First, the repeated-measures correlation (rmcorr) was computed between the TT4 and each haematological parameter. This method estimates the common within-individual slope while treating cat identity as a categorical factor, thereby removing between-cat variance from the association and preserving the independence assumption [32]. Second, linear mixed-effects models were fitted with cat identity as a random intercept, in which TT4 was partitioned into two orthogonal predictors: the cat-specific mean TT4 across the four timepoints (between-cat component) and the deviation of each observation from that cat-specific mean (within-cat component). This decomposition distinguishes the association attributable to differences between cats from the association attributable to changes occurring within the same cat over time, and it therefore tests directly whether haematological parameters track TT4 within individual animals rather than merely separating cats with divergent therapeutic outcomes. Marginal and conditional coefficients of determination were calculated according to Nakagawa and Schielzeth [33]. Random-intercept models were used. Random-slope models were not fitted, since four observations per cat provide insufficient information to estimate subject-specific slopes reliably; repeated-measures correlation likewise assumes a common within-subject slope across cats. Two additional sensitivity analyses were performed: repeated-measures correlations stratified by therapeutic response group, and repeated-measures correlations after exclusion of Group C, in which loss of control resulted from owner-initiated treatment discontinuation rather than from pharmacodynamic failure. The within-subject analyses and the sensitivity analyses were added during revision and are therefore exploratory.

The following elements were defined before data collection began: the four sampling timepoints, the parameters recorded at each visit, the primary objective (correlation between TT4 and Hct, Hgb and MCV at each timepoint), and the secondary objectives (bidirectional haematological dynamics following loss of therapeutic control, and ROC-based discrimination of uncontrolled hyperthyroidism). Categorisation of cats into therapeutic response Groups A to D could only be established once the six-month follow-up was complete and is therefore retrospective by design. Consequently, all group-stratified analyses are descriptive and exploratory. The ROC cut-offs, although derived from a pre-specified secondary objective, are data-derived and are likewise reported as exploratory and hypothesis-generating rather than confirmatory.

## 3. Results

### 3.1. Cohort Characteristics

Forty-three cats (mean age: 12.4 ± 2.2 years; 28 males [20 castrated, eight intact], 15 females [12 spayed, three intact]; mean body weight: 3.58 ± 0.57 kg) completed the six-month follow-up. Baseline TT4 was 8.31 ± 1.26 µg/dL (range: 5.4–10.8). Systolic blood pressure was 173 ± 14 mmHg; hypertension (>160 mmHg) was present in 35/43 cats (81.4%), a prevalence higher than the 10–23% reported in general hyperthyroid populations [2,9], likely reflecting the treatment-naïve and referral nature of this cohort. Baseline reticulocyte count was 49.57 ± 10.17 ×10^9^/L and SDMA was 10.95 ± 1.71 µg/dL, both within feline reference ranges. Descriptive statistics per parameter and timepoint are shown in Table 1. No cat developed clinical signs attributable to methimazole toxicity during the study period, and no cat was withdrawn from treatment because of an adverse drug reaction. Leucocyte, neutrophil and platelet counts were transcribed at T0 only; all values were within reference limits at that timepoint. Subclinical haematological adverse effects arising after T0 therefore cannot be formally excluded. Dose adjustments were required in 19/43 cats (Groups B–C): methimazole was titrated upward in eight cats (Group B) and discontinued in 11 cats (Group C) due to owner non-compliance between T1 and T2.

### 3.2. Longitudinal Evolution of Parameters

The Shapiro–Wilk test identified non-normal distributions for at least one timepoint per parameter (TT4: T1–T3; Hct: T2–T3; Hgb: T3; MCV: T0; ALT: T1 and T3), justifying the use of Friedman’s test over parametric alternatives. Friedman’s test revealed significant longitudinal changes for all five parameters: TT4 (χ^2^ = 26.17, Kendall W = 0.203, *p* < 0.001); Hct (χ^2^ = 28.86, W = 0.224, *p* < 0.001); MCV (χ^2^ = 13.48, W = 0.104, *p* = 0.004); Hgb (χ^2^ = 34.38, W = 0.266, *p* < 0.001); and ALT (χ^2^ = 31.24, W = 0.242, *p* < 0.001) (Table 2). Effect sizes, interpreted according to Cohen’s convention for Kendall’s W (small: 0.1; medium: 0.3; large: 0.5) were medium for TT4, Hct, Hgb and ALT (W = 0.203–0.266) and small for MCV (W = 0.104). Post hoc Wilcoxon tests identified significant differences at T0 vs. T1 for all parameters (all *p* < 0.001 after Bonferroni correction). At T0 vs. T2, TT4, Hct, Hgb and ALT remained significant after Bonferroni correction (all *p* ≤ 0.008); MCV did not reach significance after correction (*p* = 0.084, ns). At T0 vs. T3, significant differences were detected for Hct (*p* = 0.032) and TT4 (*p* = 0.038) but not for Hgb (*p* = 0.068, ns), MCV (*p* = 0.196, ns) or ALT (*p* = 0.290, ns; Table 2). These comparisons should not be interpreted as evidence of equivalence, and formal equivalence testing was not performed.

**Figure 1:**
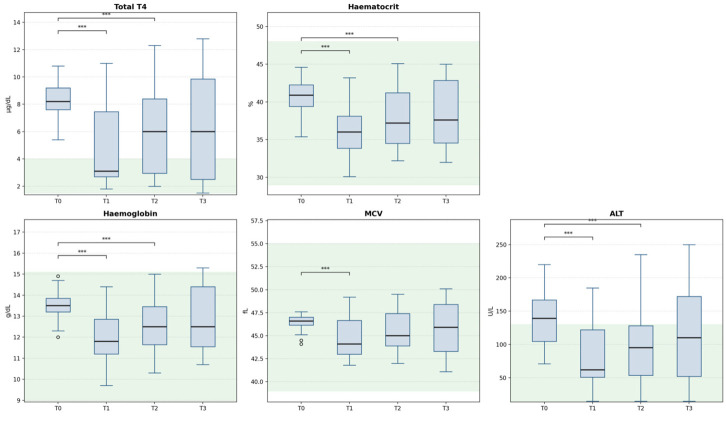
Longitudinal evolution of TT4 and haematological parameters across four timepoints (*n* = 43). Box plots show median (horizontal line), interquartile range (box) and 1.5x IQR whiskers; open circles indicate outliers. Green shading: feline reference range. *** *p* < 0.0083 vs. T0 (Bonferroni-corrected Wilcoxon signed-rank test). ALT: alanine aminotransferase; MCV: mean corpuscular volume; TT4: total serum thyroxine.

**Figure 2:**
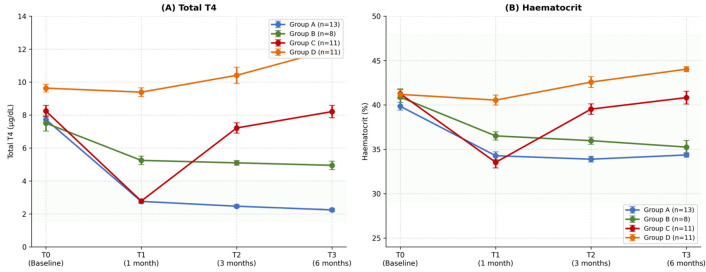
Mean ± SD of TT4 (**A**) and haematocrit (**B**) across timepoints, stratified by therapeutic response group. Group A: sustained euthyroidism (*n* = 13); Group B: partial response requiring dose adjustment (*n* = 8); Group C: initial response followed by loss of control (*n* = 11); Group D: primary non-responders (*n* = 11). Green shading: reference range.

The mean Hct decreased from 40.74 ± 1.87% at T0 to 36.10 ± 3.29% at T1 (−11.4%). A further statistically significant increase was observed at T2 (37.94 ± 3.82%, Wilcoxon *p* = 0.002 vs. T1) and T3 (38.65 ± 4.39%), driven by Group C relapse and Group D persistence, consistent with ongoing or recurrent thyroid hormone stimulation of erythropoiesis in non-euthyroid cats. The MCV decreased from 46.46 ± 0.76 fL to 44.67 ± 2.10 fL at T1. Hgb and ALT showed analogous declines from T0 to T1, with subsequent partial recovery at T2 and T3 consistent with the pattern observed for the haematocrit. No cat developed clinically significant anaemia (Hct < 25%) at any timepoint, and all haematocrit values remained within or close to the laboratory reference interval (29–48%). At baseline, no cat exceeded the upper reference limit for haematocrit (maximum: 44.6%; reference interval: 29 to 48%), MCV (maximum: 47.6 fL; reference interval: 39 to 55 fL) or haemoglobin (maximum: 14.9 g/dL; reference interval: 9.0 to 15.1 g/dL). Erythrocytic indices at presentation were therefore situated in the upper part of the reference interval rather than frankly increased, and overt erythrocytosis was not documented in this cohort. Haemoglobin showed a significant increase from T1 to T2 (11.97 ± 1.26 vs. 12.63 ± 1.21 g/dL; Wilcoxon *p* = 0.0003, surviving the Bonferroni-corrected threshold of α = 0.0083), driven primarily by Group C cats whose TT4 rebounded following methimazole discontinuation (mean Hgb increase +2.00 g/dL in Group C vs. +0.07 g/dL in Groups A–B combined), consistent with the bidirectional relationship between thyroid status and erythropoiesis in this subgroup (*n* = 11). Longitudinal box plots are shown in Figure 1 and group-stratified trajectories in Figure 2. Reticulocyte counts followed a similar trajectory: mean values declined from 49.57 ± 10.17 ×10^9^/L at T0 to 38.27 ± 10.24 ×10^9^/L at T1 (Friedman χ^2^ = 23.57, Kendall W = 0.183, *p* < 0.001). Post hoc Wilcoxon tests identified significant decreases at T0 vs. T1 (*p* = 0.002) and T0 vs. T3 (*p* = 0.001), with T0 vs. T2 reaching trend level only (*p* = 0.013; not significant after Bonferroni correction at α = 0.0083); no significant differences were detected among T1, T2 and T3 (all *p* ≥ 0.280). With the exception of 3/43 cats that marginally exceeded the upper limit at T0 (72.0, 71.5 and 63.1 × 10^9^/L), reticulocyte values remained within the feline reference range (0–60 × 10^9^/L [9]) at all timepoints, indicating that reticulocytosis was not a consistent feature of this cohort at baseline.

**Table 2:** Friedman test statistics and Wilcoxon post hoc *p*-values with Bonferroni correction (*n* = 43).

Parameter	χ^2^ (Kendall’s W)	*p* (Global)	T0 vs. T1	T0 vs. T2	T0 vs. T3	T1 vs. T2	T1 vs. T3	T2 vs. T3
Total T4 (µg/dL)	26.17 (W = 0.203)	<0.001	<0.001 ***	<0.001 ***	0.038 †	0.012 †	0.001 ***	0.098 ns
Haematocrit (%)	28.86 (W = 0.224)	<0.001	<0.001 ***	<0.001 ***	0.032 †	0.002 ***	<0.001 ***	0.036 †
Haemoglobin (g/dL)	34.38 (W = 0.266)	<0.001	<0.001 ***	<0.001 ***	0.068 ns	0.0003 ***	<0.001 ***	0.074 ns
MCV (fL)	13.48 (W = 0.104)	0.004	<0.001 ***	0.084 ns	0.196 ns	0.008 ***	0.001 ***	0.087 ns
ALT (U/L)	31.24 (W = 0.242)	<0.001	<0.001 ***	<0.001 ***	0.290 ns	<0.001 ***	<0.001 ***	0.076 ns
Reticulocytes (×10^9^/L)	23.57 (W = 0.183)	<0.001	0.002 ***	0.013 †	0.001 ***	0.671 ns	0.280 ns	0.542 ns

*** *p* < 0.0083 (significant after Bonferroni correction, 6 comparisons); † trend: 0.0083 ≤ *p* < 0.05; ns: *p* ≥ 0.05. χ^2^: Friedman chi-square; df = 3 for all tests. The T0-to-T3 difference for TT4, while reaching trend level (*p* = 0.038), reflects the partial response of the cohort as a whole, with 29/43 cats remaining uncontrolled at T3.

### 3.3. Correlation Between TT4 and Haematological Parameters

**Figure 3:**
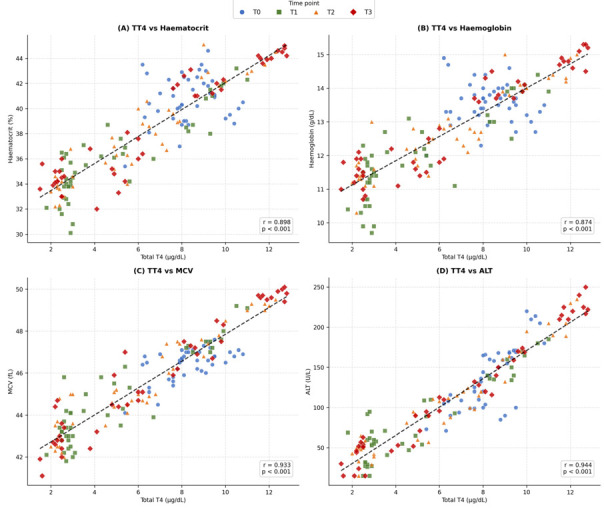
Pearson correlations between TT4 and haematological parameters (pooled *n* = 172). (**A**) Haematocrit; (**B**) haemoglobin; (**C**) MCV; (**D**) ALT. Data points are colour-coded by timepoint; dashed line: linear regression. Note that T0 data points form a distinct cluster in the upper-right quadrant, reflecting restricted TT4 variability at baseline; the full dynamic range emerges at T1–T3 as treatment separates responders from non-responders. r: Pearson coefficient (all *p* < 0.001 for pooled data). ALT: alanine aminotransferase; MCV: mean corpuscular volume.

Pearson correlations were calculated separately at each timepoint (*n* = 43 per timepoint) to avoid pseudo-replication inherent in pooled repeated-measures data. Complete results with *p*-values are presented in Table 3. At T0, correlations with Hct (r = 0.186, *p* = 0.232) and Hgb (r = −0.067, *p* = 0.670) were non-significant, reflecting limited TT4 variability when all cats shared similarly elevated baseline values. MCV (r = 0.594, *p* < 0.001) and ALT (r = 0.782, *p* < 0.001) were already significant at T0. For MCV, this likely reflects the fact that erythrocyte volume is determined by individual cell size rather than total erythrocyte mass, making it less dependent on haemodynamic variables such as hydration status and thus more sensitive to the direct effect of thyroid hormones on erythroid maturation even before treatment separates responders from non-responders. ALT was already significantly elevated at T0, consistent with active hepatic involvement proportional to thyroid hormone excess at presentation. From T1 onwards, all primary haematological parameters (Hct, Hgb, MCV) and ALT reached significance (all *p* ≤ 0.0003). Correlations intensified progressively from T1 to T3: Hct (r = 0.875 → 0.943); Hgb (r = 0.836 → 0.946); MCV (r = 0.907 → 0.964); and ALT (r = 0.919 → 0.981) (Table 3; Figure 3). Leucocyte count was assessed at T0 only (r = 0.658, *p* < 0.001) and is reported in the text; longitudinal data were not available and are therefore excluded from Table 3. Reticulocyte count showed a significant moderate correlation at T0 (r = 0.369, *p* = 0.015, not surviving Bonferroni correction), a non-significant correlation at T1 (r = 0.003, *p* = 0.984), a significant moderate correlation at T2 (r = 0.486, *p* < 0.001) and a significant strong correlation at T3 (r = 0.744, *p* < 0.001). The pooled correlation was r = 0.490 (r^2^ = 0.240, *p* < 0.001). These results are detailed in Table 3. While pooled correlations for the primary parameters may partially reflect the bimodal TT4 distribution across treatment groups, the progressive strengthening of per-timepoint correlations from T0 (Hct r = 0.186) to T3 (Hct r = 0.943) argues against a purely distributional artefact and supports a plausible functional relationship that intensifies as therapeutic divergence accumulates.

**Table 3:** Pearson and Spearman correlation coefficients between TT4 and haematological parameters: pooled (*n* = 172) and per timepoint (*n* = 43 each).

Parameter	r (Pooled, Ref. Only)	r^2^	*p*	r at T0	r at T1	r at T2	r at T3
Haematocrit (%)	0.898	0.807	<0.001	0.186	0.875	0.935	0.943
Spearman ρ	–	–	–	0.123	0.789	0.923	0.892
Haemoglobin (g/dL)	0.874	0.764	<0.001	−0.067	0.836	0.908	0.946
Spearman ρ	–	–	–	−0.105	0.771	0.869	0.878
MCV (fL)	0.933	0.87	<0.001	0.594	0.907	0.944	0.964
Spearman ρ	–	–	–	0.561	0.787	0.916	0.94
ALT (U/L)	0.944	0.892	<0.001	0.782	0.919	0.96	0.981
Spearman ρ	–	–	–	0.753	0.779	0.935	0.966
Reticulocytes (×10^9^/L)	0.49	0.24	<0.001	0.369	0.003	0.486	0.744
Spearman ρ	–	–	–	0.268	−0.035	0.491	0.765

r: Pearson correlation coefficient; r^2^: coefficient of determination. Per-timepoint correlations (r at T0 to T3) are the primary analyses; pooled correlations (*n* = 172, 43 cats × 4 timepoints) are presented for reference only and should be interpreted with caution given the non-independence of repeated observations within subjects. All pooled *p* < 0.001. Individual timepoint correlations significant at *p* < 0.05 except haematocrit at T0 (r = 0.186, *p* = 0.232) and haemoglobin at T0 (r = −0.067, *p* = 0.670). Spearman rank correlation coefficients (ρ) are reported for every timepoint alongside Pearson’s r. Pooled Spearman coefficients are not reported, since pooled analyses violate the independence assumption and are presented for reference only. The Spearman ρ at T0 was non-significant for haematocrit (ρ = 0.123, *p* = 0.434), haemoglobin (ρ = −0.105, *p* = 0.505) and reticulocytes (ρ = 0.268, *p* = 0.082) and significant for MCV (ρ = 0.561, *p* < 0.001) and ALT (ρ = 0.753, *p* < 0.001). At T1, the Spearman ρ was non-significant for reticulocytes (ρ = −0.035, *p* = 0.822). All other Spearman coefficients were significant at *p* < 0.001.

### 3.4. Within-Subject Tracking of TT4

Because per-timepoint correlations cannot distinguish an association driven by differences between cats from an association driven by changes within the same cat, two within-subject analyses were performed (Table 4).

Repeated-measures correlation demonstrated a strong common within-individual association between the TT4 and each primary erythrocytic parameter: Hct (rmcorr r = 0.873, 95% CI 0.830 to 0.910, df = 128, *p* < 0.001); MCV (rmcorr r = 0.881, 95% CI 0.840 to 0.910, *p* < 0.001); and Hgb (rmcorr r = 0.865, 95% CI 0.810 to 0.900, *p* < 0.001). ALT showed the strongest within-individual association (rmcorr r = 0.900, 95% CI 0.860 to 0.930, *p* < 0.001), whereas reticulocytes showed only a weak association (rmcorr r = 0.309, 95% CI 0.140 to 0.460, *p* < 0.001).

Linear mixed-effects models with cat identity as a random intercept confirmed these findings and allowed the within-cat and between-cat components of the TT4 association to be quantified separately. For haematocrit, each 1 µg/dL increase in TT4 relative to a cat’s own mean was associated with an increase of 1.235 percentage points (95% CI 1.116 to 1.354, *p* < 0.001), whereas the between-cat coefficient was 0.979 (95% CI 0.867 to 1.091, *p* < 0.001). The corresponding within-cat coefficients were 0.651 fL for MCV (95% CI 0.591 to 0.712, *p* < 0.001), 0.428 g/dL for Hgb (95% CI 0.385 to 0.471, *p* < 0.001) and 16.95 U/L for ALT (95% CI 15.53 to 18.38, *p* < 0.001). Marginal coefficients of determination ranged from 0.780 for Hgb to 0.891 for ALT. For haematocrit and haemoglobin, the within-cat coefficient exceeded the between-cat coefficient, indicating that the association is not merely a reflection of separation between therapeutic response groups but reflects changes occurring within individual cats over time.

Two sensitivity analyses supported this interpretation. First, repeated-measures correlations remained significant within each therapeutic response group analysed separately, including Group A, in which all cats remained euthyroid at every post-treatment visit and no contrast between controlled and uncontrolled states was available (Hct rmcorr r = 0.926; MCV rmcorr r = 0.889; Hgb rmcorr r = 0.904; all *p* < 0.001). The corresponding values were 0.841, 0.625 and 0.829 in Group B, 0.938, 0.947 and 0.905 in Group C, and 0.688, 0.863 and 0.746 in Group D (all *p* < 0.001). Second, after exclusion of the 11 Group C cats, in whom loss of control followed owner-initiated treatment discontinuation, the within-individual associations persisted in the remaining 32 cats (Hct rmcorr r = 0.831; MCV rmcorr r = 0.845; Hgb rmcorr r = 0.838; all *p* < 0.001), indicating that the observed tracking does not depend on the treatment interruption subgroup.

### 3.5. Bidirectional Dynamics in Group C

**Figure 4:**
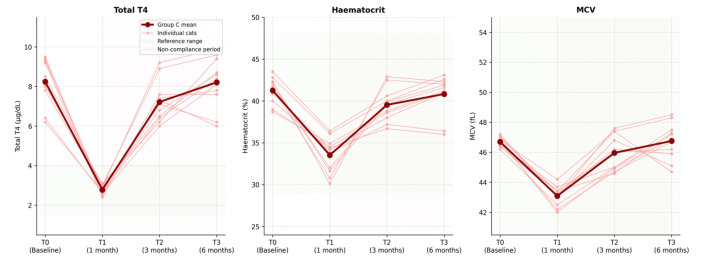
Bidirectional dynamics of TT4, haematocrit and MCV in Group C (*n* = 11). Thin lines: individual cats; thick line: group mean. Red shading: period of non-compliance and methimazole discontinuation (between T1 and T2). Green shading: feline reference range.

In Group C (*n* = 11), initial TT4 normalisation at T1 (mean 2.78 ± 0.22 µg/dL) was accompanied by Hct and MCV decreases consistent with Groups A and B. Following methimazole discontinuation due to owner non-compliance between T1 and T2, the TT4 rose to 7.22 ± 1.04 µg/dL at T2 and to 8.21 ± 1.27 µg/dL at T3, with parallel increases in the Hct (39.5 ± 2.0% at T2) and MCV (46.0 ± 1.2 fL at T2) towards pre-treatment levels. These bidirectional longitudinal changes, specifically normalisation followed by return to hyperthyroid values, are illustrated in Figure 4. The Wilcoxon signed-rank test within Group C confirmed that the reversal between T1 and T2 was statistically significant for TT4 (*p* < 0.001), haematocrit (*p* < 0.001) and MCV (*p* < 0.001), providing formal statistical evidence of bidirectional haematological dynamics in response to loss of therapeutic control. From a methodological perspective, Group C constitutes an unplanned withdrawal–rechallenge natural experiment: treatment discontinuation was owner-initiated and unforeseeable at study design yet produced a controlled condition that would be ethically impossible to generate intentionally. The parallel bidirectional response of the haematological parameters in this subgroup therefore provides supportive observational evidence beyond what cross-sectional or purely unidirectional longitudinal designs could offer. Reticulocyte counts in Group C did not show a consistent bidirectional pattern at group level (mean T0: 49.1 ± 6.2, T1: 38.8 ± 11.6, T2: 37.0 ± 13.5, T3: 46.5 ± 8.8 × 10^9^/L), with substantial inter-individual variability precluding group-level conclusions.

### 3.6. Renal Function Monitoring

SDMA remained within the feline reference range (≤14 µg/dL) at all four timepoints for all 43 cats (mean T0: 10.95 ± 1.71 µg/dL; T1: 10.88 ± 1.97 µg/dL; T2: 11.00 ± 1.92 µg/dL; T3: 11.28 ± 1.97 µg/dL). No significant longitudinal change was detected (Friedman χ^2^ = 6.64, Kendall W = 0.051, *p* = 0.084), and a weak but statistically significant pooled correlation was identified between SDMA and TT4 (pooled r = 0.195, *p* = 0.012) and between SDMA and haematocrit (r = 0.244, *p* = 0.001); the magnitude of these associations does not suggest clinically meaningful confounding of the TT4–haematocrit relationship (combined r^2^ values explain less than 6% of Hct variance), supporting the conclusion that renal function remained stable throughout the study period.

### 3.7. ROC Analysis

**Figure 5:**
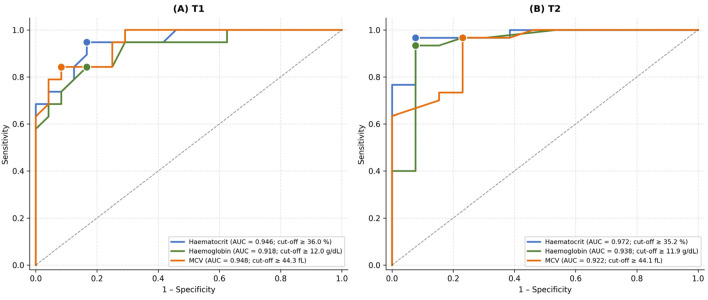
ROC curves for detection of uncontrolled hyperthyroidism (TT4 > 4.0 µg/dL) using haematological parameters at T1 (**A**) and T2 (**B**). Filled circles: optimal cut-off points (Youden index). AUC: area under the curve; MCV: mean corpuscular volume.

ROC analysis was performed at T1 and T2, the clinically most relevant monitoring visits; T3 was excluded because haematological cut-offs derived at T2 would apply identically at T3, given the near-identical distribution of controlled and uncontrolled cases (T2: 30/43 uncontrolled, 69.8%; T3: 29/43 uncontrolled, 67.4%), which would not constitute independent validation of the T2-derived thresholds. Discriminatory cut-offs were identified for all three haematological parameters at both T1 and T2 (Table 5; Figure 5). At T2, Hct ≥ 35.2% yielded AUC = 0.972 (95% CI bootstrap 0.914–1.000; sensitivity 96.7%, specificity 92.3%; Youden index 0.890). Haemoglobin ≥ 11.9 g/dL at T2 achieved AUC = 0.938 (95% CI bootstrap 0.821–1.000; sensitivity 93.3%, specificity 92.3%). At T1, Hct ≥ 36.0% yielded AUC = 0.946 (95% CI bootstrap 0.869–0.993; sensitivity 94.7%, specificity 83.3%), suggesting that haematological discrimination is already potentially meaningful at the first monitoring visit. The wide bootstrap confidence intervals across all parameters (ranging from 0.812–0.986 for Hgb at T1 to 0.914–1.000 for Hct at T2) reflect the modest sample size and should be interpreted with appropriate caution; the proposed exploratory cut-offs remain preliminary and require external validation.

**Table 5:** ROC analysis for detection of uncontrolled hyperthyroidism (TT4 > 4.0 µg/dL) at T1 and T2.

Timepoint	Parameter	AUC (95% CI)	Optimal Cut-Off (Youden)	Sensitivity (%)	Specificity (%)	Youden Index
T1	Haematocrit (%)	0.946 (0.869–0.993)	≥36.0%	94.7	83.3	0.781
	MCV (fL)	0.948 (0.877–0.993)	≥44.3 fL	84.2	91.7	0.759
	Haemoglobin (g/dL)	0.918 (0.812–0.986)	≥12.0 g/dL	84.2	83.3	0.675
T2	Haematocrit (%)	0.972 (0.914–1.000)	≥35.2%	96.7	92.3	0.89
	Haemoglobin (g/dL)	0.938 (0.821–1.000)	≥11.9 g/dL	93.3	92.3	0.856
	MCV (fL)	0.922 (0.821–0.994)	≥44.1 fL	96.7	76.9	0.736

AUC: area under the ROC curve. Cut-off: Youden index (J = sensitivity + specificity − 1). Outcome: TT4 > 4.0 µg/dL. T1: *n* = 19 uncontrolled/24 controlled; T2: *n* = 30 uncontrolled/13 controlled.

## 4. Discussion

This prospective longitudinal study systematically characterises the bidirectional relationship between TT4 and haematological parameters during methimazole therapy and after treatment interruption and suggests that the haematocrit and MCV at three months may discriminate between controlled and uncontrolled hyperthyroidism with promising but preliminary accuracy.

The strong correlations between the TT4 and erythrocytic parameters at T2 and T3 (Hct: r = 0.935–0.943; MCV: r = 0.944–0.964; Hgb: r = 0.908–0.946) are consistent with the well-established role of thyroid hormones in erythropoiesis, mediated through erythropoietin upregulation, sensitisation of erythroid progenitors and acceleration of erythrocyte maturation [21,22,23]. In human thyroid disease, macrocytosis and erythrocytosis accompany hyperthyroidism, while hypothyroidism produces normocytic or microcytic anaemia [24,25]. In terms of feline patients, Gójska-Zygner et al. reported erythrocytosis in 12/88 hyperthyroid cats [26], while Gil-Morales et al. reported microcytosis in 29.5% of cats referred for radioiodine [27]. The present cohort did not exhibit microcytosis at any timepoint; the MCV remained within the feline laboratory reference range throughout (39–55 fL [9]), consistent with a population presenting with moderate hyperthyroidism in which hepcidin-mediated iron restriction, the proposed mechanism linking thyroid hormone excess to microcytosis, may not yet have reached the threshold required to produce frank microcytosis [28,29], as reported in more advanced or chronic radioiodine referral cohorts [34]. To the best of our knowledge, no published study has characterised the longitudinal kinetics of the haematocrit or MCV during methimazole therapy in cats; the present study provides this characterisation prospectively for the first time. Our findings extend these cross-sectional observations to a longitudinal framework, suggesting that haematological response may mirror TT4 dynamics both during successful treatment and upon relapse.

The progressive intensification of TT4–Hct (r = 0.875 at T1 → 0.943 at T3) and TT4–MCV (r = 0.907 → 0.964) correlations, alongside the non-significant baseline correlations for Hct and Hgb at T0, may reflect increasing therapeutic divergence between responders (Groups A–B) and non-responders or relapsing cats (Groups C–D). At baseline, all cats share similarly elevated TT4, limiting correlation power; as treatment proceeds and groups separate, the haematological signal may become progressively more discriminative, a pattern reflected in the superior ROC performance at T2 compared to T1.

The within-subject analyses address this concern directly. Repeated-measures correlation, which removes between-cat variance, confirmed a strong common within-individual association between the TT4 and Hct (rmcorr r = 0.873), MCV (rmcorr r = 0.881) and Hgb (rmcorr r = 0.865). In the mixed-effects models, the within-cat coefficient for the haematocrit (1.235 percentage points per µg/dL) exceeded the between-cat coefficient (0.979), and the same pattern was observed for haemoglobin. The association therefore cannot be attributed solely to the separation of therapeutic response groups. This interpretation is reinforced by the persistence of significant within-individual associations inside Group A, in which every cat remained euthyroid at all post-treatment visits and no controlled-versus-uncontrolled contrast was available, and by the persistence of the associations after exclusion of Group C.

An important qualification must be stated explicitly. The bidirectional evidence presented here derives predominantly from cats in Group C, in whom the rise in TT4 followed owner-initiated discontinuation of methimazole rather than a controlled reduction in dose during continued therapy. The rebound observed in this subgroup therefore reflects withdrawal of treatment, not dose titration under ongoing therapeutic management, and the two situations should not be equated. Whether the haematocrit and MCV track the smaller TT4 fluctuations that occur during dose adjustment in continuously treated cats was not tested in this study and remains to be established. The sensitivity analysis excluding Group C nevertheless showed that the within-individual association persists in the remaining 32 cats, indicating that haematological tracking is not confined to the treatment interruption scenario.

The decrease in the haematocrit following methimazole should not be automatically interpreted as drug-induced anaemia but rather as the physiological resolution of thyroid hormone-driven erythropoietic stimulation. In the present cohort, no cat exceeded the upper reference limit for haematocrit, MCV or haemoglobin at baseline, so the term erythrocytosis is not applicable to these animals. Hyperthyroid cats characteristically present with Hct and MCV values in the upper part of the reference range, reflecting chronic T4- and T3-mediated erythropoietic stimulation that may remain below the threshold of overt erythrocytosis [21,22,23]. Treatment-induced TT4 normalisation removes this stimulus, gradually reducing the Hct toward mid-reference values. No cat in this cohort reached a Hct < 25% at any timepoint, supporting the clinical benignity of this shift. Furthermore, the absence of compensatory reticulocytosis during haematocrit decline argues against an acute haemolytic or cytotoxic mechanism, as drug-induced haemolytic anaemia would be expected to trigger erythropoietic rebound within days; the observed gradual decline over 30 days is instead consistent with the normal feline erythrocyte lifespan of approximately 70 days [9] and the progressive resolution of thyroid hormone-driven erythropoietic stimulation. This is distinct from the rare idiosyncratic immune-mediated haemolytic anaemia and bone marrow dyscrasia associated with methimazole toxicity, which present acutely with concurrent leucopoenia or thrombocytopenia, typically within the first four to six weeks [9,13].

The strong TT4–ALT correlation (r = 0.919 at T1, rising to r = 0.981 at T3) is consistent with previously described hepatic involvement in feline hyperthyroidism, attributed to hepatocellular hypoxia, accelerated hepatic metabolism and possible direct thyroid hormone toxicity [35,36]. Although ALT lacks thyroidal specificity, its parallel trajectory with TT4 suggests a potential role in composite monitoring panels.

Reticulocyte counts declined significantly from T0 to T1 (*p* = 0.002), consistent with the expected reduction in erythropoietic drive following TT4 normalisation under methimazole. However, reticulocyte counts remained within the feline reference range at all timepoints, indicating that the erythropoietic stimulation present at baseline, while biologically active, did not produce frank reticulocytosis in this cohort.

Furthermore, correlations between TT4 and reticulocyte count were weak at T0 (r = 0.369, not significant after Bonferroni correction) and non-significant at T1 (r = 0.003). The near-zero correlation at T1 indicates that the pre-specified hypothesis of reticulocyte tracking was not confirmed at the first post-treatment visit, likely reflecting a lag between EPO suppression and peripheral reticulocyte clearance within the approximately 70-day feline erythrocyte lifespan. Correlations strengthened only at T3 (r = 0.744), and bidirectional dynamics in Group C were inconsistent at the individual level. Collectively, these observations suggest that reticulocytes are a less reliable surrogate monitoring indicator than Hct or MCV in this population, likely because reticulocyte kinetics reflect acute erythropoietic changes occurring over days rather than the cumulative erythrocyte mass changes captured by the haematocrit and MCV over weeks to months. Reticulocyte counts may be more informative during the acute phase immediately following treatment initiation, where an early rebound would signal erythropoietic reconstitution; this hypothesis was not directly testable within the monthly sampling intervals of the present study and warrants dedicated investigation.

ROC analysis suggested Hct ≥ 35.2% at T2 as the most discriminative exploratory cut-off (AUC 0.972, 95% CI bootstrap 0.914–1.000; sensitivity 96.7%, specificity 92.3%). At T1, Hct ≥ 36.0% already yielded an AUC of 0.946 (95% CI 0.869–0.993), confirming potentially meaningful discrimination from the first monitoring visit. These point estimates are promising; bootstrap optimism correction (2000 iterations) yielded negligible optimism estimates for all parameters (all <0.003), confirming that the AUC estimates are not artefactually inflated by overfitting to the training data (a common concern when cut-offs are derived from the same dataset used for evaluation) and that the observed discriminatory performance is unlikely to represent a chance finding specific to this sample.

Nevertheless, the bootstrap confidence intervals remain wide, reflecting the modest sample size, and all estimates should be interpreted as preliminary pending external validation [30]. Practically, a haematocrit and MCV above approximately 36% and 44.3 fL, respectively, in a methimazole-treated cat may prompt a clinical re-evaluation of the therapeutic compliance and dose adequacy, while falling Hct and MCV values toward mid-reference values may reassure the clinician of ongoing control. These cut-offs are exploratory and should be contextualised against individual baseline values, hydration status and comorbidities, as well as validated prospectively before integration into clinical monitoring protocols. Importantly, Hct ≥ 35.2% falls within the normal feline reference range and would not trigger an alert on a standard haemogram without longitudinal comparison to each patient’s own pre-treatment baseline; the clinical utility of this threshold therefore depends entirely on the availability of a prior Hct measurement from the same individual. Accordingly, clinicians are encouraged to record a baseline complete blood count at T0 alongside TT4, specifically to enable longitudinal haematological comparison at subsequent monitoring visits. Leucocyte count (WBC) showed only moderate correlation with baseline TT4 (r = 0.658 at T0, *p* < 0.001), reflecting the indirect stress leukogram mechanism rather than direct myelostimulation [9,13,37], and is not recommended as a primary monitoring marker. Similarly, non-erythrocytic lineages appear largely unaffected by thyroid hormone excess: platelet function has been shown to remain within normal limits in hyperthyroid cats despite markedly elevated TT4 concentrations [37].

### Limitations

Several limitations must be acknowledged when interpreting these findings.

Sample size. With 43 cats enrolled, this study is underpowered for formal between-group statistical comparisons. Subgroup analyses (Groups A–D) are descriptive only. The sample size precludes multivariate modelling to adjust for potential confounders, such as sex, breed, disease duration at diagnosis or baseline disease severity. The absence of a euthyroid control group limits external comparability, although the within-subject design helps reduce inter-individual confounding by using each cat as its own control across four timepoints. Longitudinal leucocyte, neutrophil and platelet data were not transcribed into the study database and could not be retrieved retrospectively. Consequently, the longitudinal behaviour of the stress leukogram could not be characterised, and subclinical haematological adverse effects of methimazole arising after T0 cannot be formally excluded.

ROC confidence intervals. Bootstrap 95% confidence intervals for AUC values were calculated (2000 iterations) and are reported in the Results and Table 5. The intervals are wide, ranging from 0.869–0.993 for Hct at T1 to 0.914–1.000 for Hct at T2, reflecting the current sample size (*n* = 43). Although the true AUC in a larger population could differ from the point estimate, the consistency of the discriminatory performance across two independent monitoring visits (T1 and T2) reduces the probability that the observed AUC values represent chance findings. Bootstrap optimism correction yielded negligible optimism for all parameters (all <0.003), confirming the absence of meaningful overfitting on the present dataset. The proposed cut-offs should be regarded as preliminary and hypothesis-generating, requiring prospective external validation before clinical adoption. Based on the Hanley–McNeil method, a validation study designed to confirm AUC ≥ 0.80 for Hct at T2 (80% power, α = 0.05) would require approximately 40 cats with at least 19 in each outcome class; the current dataset (30 uncontrolled and 13 controlled at T2) already meets this threshold for the majority class, supporting the internal stability of the AUC estimates.

Single-centre design. This study was conducted at a single referral institution (USAMV Cluj-Napoca), which may limit generalisability to primary-care or geographically diverse populations with different disease prevalence and case-mix characteristics. External validation in a multicentre setting is required before the proposed cut-offs can be broadly applied.

Cohort selection. Cats with CKD IRIS stage 2 or higher, neoplasia, inflammatory disease or anaemia of another aetiology were excluded in order to isolate the relationship between thyroid status and erythropoiesis. These comorbidities are common in geriatric hyperthyroid cats in routine practice, and the screening-to-enrolment ratio of approximately 3:1 confirms that a substantial proportion of screened cats did not meet the eligibility criteria. The cohort is therefore highly selected and does not represent the broader population of hyperthyroid cats encountered in primary-care or referral settings. The proposed haematological cut-offs may not be transferable to cats with concurrent chronic kidney disease, chronic inflammation or neoplasia, all of which independently affect erythrocyte indices, and their clinical application should, for the present, be restricted to cats in whom such comorbidities have been excluded.

Disease prevalence and predictive values. The proportion of uncontrolled cats at T2 was 69.8% (30/43), substantially higher than would be expected in a primary-care setting where compliant patients predominate. This high prevalence, partly attributable to owner non-compliance in Group C and primary non-response in Group D, inflates the positive predictive value of the haematological cut-offs derived here. In populations with lower prevalence of uncontrolled hyperthyroidism, the positive predictive value will be lower and the negative predictive value higher; prospective validation in primary-care settings is therefore required before these cut-offs can be broadly applied.

Haematological confounders. The haematocrit is influenced by hydration status, plasma volume shifts and the venipuncture-related stress haemoconcentration, none of which were systematically controlled in this study. In geriatric cats with concurrent hypertension, present in 81.4% of this cohort, plasma volume fluctuations may independently affect the Hct between timepoints [38]. Total protein, albumin, urea, and creatinine, urine-specific gravity and a quantitative assessment of hydration were not recorded systematically, and this is a limitation of the present study. Hydration status was assessed clinically at every visit as part of the routine physical examination, and no cat was considered clinically dehydrated at any timepoint, but this assessment was not quantified. No cat was sedated for venipuncture, which removes sedation-related haemodynamic shifts as a confounder. Two observations argue against the haemoconcentration as the principal driver of the observed dynamics. First, the MCV reflects the individual erythrocyte volume and is substantially less influenced by hydration status or plasma volume shifts than the haematocrit, yet the MCV and Hct moved in parallel at every timepoint; a purely haemoconcentration-driven artefact would not be expected to produce concordant MCV changes. Second, SDMA remained within the reference interval in all 43 cats at all four timepoints, with no significant longitudinal change, arguing against substantial renally mediated shifts in plasma volume. These arguments reduce, but do not eliminate, the possibility that hydration and the stress-related haemoconcentration contributed to the observed haematocrit changes, and the haematocrit results should be interpreted with this reservation.

## 5. Conclusions

This study suggests that the haematocrit and MCV changed bidirectionally with the TT4 during six months of methimazole therapy and after treatment interruption in hyperthyroid cats. The major haematological shift occurs within the first 30 days and is generally sustained in well-controlled patients, reflecting the physiological resolution of thyroid hormone-driven erythropoietic stimulation rather than drug-induced anaemia. In cats whose treatment was interrupted by their owners, the haematocrit and MCV values returned toward pre-treatment values in parallel with rising TT4. This bidirectional evidence derives from treatment discontinuation rather than from dose titration during continued therapy, and the ability of haematological parameters to track the smaller TT4 fluctuations occurring during dose adjustment remains to be established. The strength of TT4–haematocrit and TT4-MCV correlations intensified progressively from T1 to T3 (Hct: r = 0.875 rising to 0.943; MCV: r = 0.907 rising to 0.964), while baseline correlations for Hct and Hgb were non-significant, indicating that haematological discrimination between controlled and uncontrolled patients improves as therapeutic divergence accumulates over time. Within-subject analyses confirmed that this association reflects changes occurring within individual cats and not merely the separation of therapeutic response groups: repeated-measures correlations were 0.873 for the haematocrit and 0.881 for the MCV, and in the mixed-effects models, the within-cat coefficient for the haematocrit exceeded the between-cat coefficient. Preliminary ROC analysis suggested a haematocrit at or above 35.2% at three months as the most discriminative exploratory cut-off for detecting uncontrolled hyperthyroidism (AUC = 0.972, 95% CI bootstrap 0.914 to 1.000; sensitivity 96.7%, specificity 92.3%), though the wide confidence interval reflects the modest sample size and mandates external validation. These cut-offs should always be interpreted in relation to each individual patient’s own pre-treatment baseline. Reticulocyte counts declined significantly from T0 to T1 but did not demonstrate consistent bidirectional tracking and are not recommended as primary surrogate monitoring indicators. Collectively, these findings support the use of the complete blood count as a low-cost complementary monitoring signal between scheduled TT4 measurements, without replacing endocrine testing. Prospective multicentre validation is required before formal clinical recommendations can be issued.

## Figures and Tables

**Table 1:** Descriptive statistics of TT4 and haematological parameters across four timepoints (*n* = 43).

Parameter	Timepoint	*n*	Mean ± SD	Median	Min	Max
Total T4 (µg/dL)	T0	43	8.31 ± 1.26	8.2	5.4	10.8
	T1	43	4.93 ± 2.87	3.1	1.8	11
	T2	43	6.20 ± 3.23	6	2	12.3
	T3	43	6.77 ± 3.91	6	1.5	12.8
Haematocrit (%)	T0	43	40.74 ± 1.87	40.9	35.4	44.6
	T1	43	36.10 ± 3.29	36	30.1	43.2
	T2	43	37.94 ± 3.82	37.2	32.2	45.1
	T3	43	38.65 ± 4.39	37.6	32	45
Haemoglobin (g/dL)	T0	43	13.54 ± 0.60	13.5	12	14.9
	T1	43	11.97 ± 1.26	11.8	9.7	14.4
	T2	43	12.63 ± 1.21	12.5	10.3	15
	T3	43	12.92 ± 1.53	12.5	10.7	15.3
MCV (fL)	T0	43	46.46 ± 0.76	46.6	44.1	47.6
	T1	43	44.67 ± 2.10	44.1	41.8	49.2
	T2	43	45.52 ± 2.25	45	42	49.5
	T3	43	45.90 ± 2.80	45.9	41.1	50.1
ALT (U/L)	T0	43	137.88 ± 38.98	139	71	220
	T1	43	81.77 ± 49.00	62	15	185
	T2	43	104.70 ± 58.71	95	15	235
	T3	43	116.88 ± 73.39	110	15	250
Reticulocytes (×10^9^/L)	T0	43	49.57 ± 10.17	48.7	23.7	72
	T1	43	38.27 ± 10.24	37	20.2	63.1
	T2	43	40.49 ± 12.24	38.4	18	66.7
	T3	43	42.94 ± 9.45	43.5	26.9	59.7
SDMA (µg/dL)	T0	43	10.95 ± 1.71	11.6	6.7	14
	T1	43	10.88 ± 1.97	11.5	6.8	13.4
	T2	43	11.00 ± 1.92	11.1	6	13.3
	T3	43	11.28 ± 1.97	11	7.3	13.8

SD: standard deviation; TT4: total serum thyroxine; MCV: mean corpuscular volume; ALT: alanine aminotransferase. T0: baseline; T1: 30 days; T2: 90 days; T3: 180 days post-treatment initiation.

**Table 4:** Within-subject association between TT4 and haematological parameters: repeated-measures correlation and linear mixed-effects models (43 cats, 172 observations).

Parameter	rmcorr r (95% CI)	Within-Cat β (95% CI)	Between-Cat β (95% CI)	R^2^m	R^2^c
Haematocrit (%)	0.873 (0.830 to 0.910)	+1.235 (1.116 to 1.354)	+0.979 (0.867 to 1.091)	0.816	0.835
Haemoglobin (g/dL)	0.865 (0.810 to 0.900)	+0.428 (0.385 to 0.471)	+0.320 (0.274 to 0.366)	0.780	0.822
MCV (fL)	0.881 (0.840 to 0.910)	+0.651 (0.591 to 0.712)	+0.635 (0.586 to 0.684)	0.868	0.871
ALT (U/L)	0.900 (0.860 to 0.930)	+16.95 (15.53 to 18.38)	+17.99 (16.65 to 19.33)	0.891	0.903
Reticulocytes (×10^9^/L)	0.309 (0.140 to 0.460)	not modelled	not modelled	–	–

rmcorr: repeated-measures correlation; df = 128 for all parameters; all *p* < 0.001. Within-cat β: change in the parameter per 1 µg/dL deviation of the TT4 from the cat’s own mean. Between-cat β: change in the parameter per 1 µg/dL difference in the cat-specific mean TT4. R^2^m: marginal coefficient of determination (fixed effects only); R^2^c: conditional coefficient of determination (fixed and random effects), calculated according to Nakagawa and Schielzeth [33]. The mixed-effects model for reticulocytes did not converge reliably and is therefore not reported.

## Data Availability

The raw data supporting the conclusions of this article will be made available by the authors on request.

## Abbreviations

The following abbreviations are used in this manuscript:

ALT	Alanine aminotransferase
AUC	Area under the curve
CKD	Chronic kidney disease
ELISA	Enzyme-linked immunosorbent assay
Hct	Haematocrit
Hgb	Haemoglobin
IRIS	International Renal Interest Society
MCHC	Mean corpuscular haemoglobin concentration
MCV	Mean corpuscular volume
Retic	Reticulocyte count
rmcorr	Repeated-measures correlation
SDMA	Symmetric dimethylarginine
ROC	Receiver operating characteristic
T4	Thyroxine
TT4	Total serum thyroxine
USAMV	University of Agricultural Sciences and Veterinary Medicine
WBC	White blood cell count
